# What Egyptians think. Knowledge, attitude, and opinions of Egyptian patients towards biobanking issues

**DOI:** 10.1186/s12910-019-0394-6

**Published:** 2019-08-09

**Authors:** Ahmed S. Abdelhafiz, Eman A. Sultan, Hany H. Ziady, Ebtesam Ahmed, Walaa A. Khairy, Douaa M. Sayed, Rana Zaki, Merhan A. Fouda, Rania M. Labib

**Affiliations:** 1 Department of Clinical pathology, National Cancer Institute, Cairo University, Kasr Al-Aini Street, Fom Elkhalig square, Cairo, 11796 Egypt; 2 Department of Community Medicine, Faculty of Medicine, Alexandria University, Alexandria, Egypt; 3 College of Pharmacy and Health Sciences, St. John’s University, New York, NY USA; 4 Department of Community Medicine, Faculty of Medicine, Cairo University, Cairo, Egypt; 5 Department of Clinical pathology, South Egypt Cancer Institute, Assiut University, Assiut, Egypt; 6 Faculty of Medicine, Cairo University, Cairo, Egypt; 7 Research Department, Children’s Cancer Hospital Egypt, 57357 Cairo, Egypt

**Keywords:** Biobanking, Egypt, Patients, Knowledge, Attitude, Opinions

## Abstract

**Background:**

Biobanking is a relatively new concept in Egypt. Building a good relationship with different stakeholders is essential for the social sustainability of biobanks. To establish this relationship, it is necessary to assess the attitude of different groups towards this concept. The objective of this work is to assess the knowledge, attitude, and opinions of Egyptian patients towards biobanking issues.

**Methods:**

We designed a structured survey to be administered to patients coming to the outpatient clinics in 3 university hospitals in Egypt. The survey included questions estimating the level of knowledge about the term “Biobank”, together with questions about the attitudes and opinions about related issues.

**Results:**

Two hundred and fifty-nine patients participated in the survey. Eighty-one percent of participants reported that they never heard about the term before. About 85% expressed that they would be willing to donate their samples for research and about 87% thought that sample donation did not contradict their religious beliefs. Fifty eight percent were willing to participate in a genetic research project, 27.8% supported sharing their sample with pharmaceutical companies, and 32.4% agreed to share their samples with institutions abroad.

**Conclusion:**

Although there is limited knowledge about biobanking among Egyptian patients, many had a positive attitude towards sample donation and didn’t show religious concerns against it. However, they showed concerns regarding participation in genetic research and with sharing their samples across borders or with pharmaceutical companies. Public education about biobanking is possible, taking into consideration the specific cultural and legal framework in Egypt.

**Electronic supplementary material:**

The online version of this article (10.1186/s12910-019-0394-6) contains supplementary material, which is available to authorized users.

## Background

Recent advances in biomedical field have increased the value of research on stored biological samples in biobanks to aid in the development of genetic-related research in many developing countries. Such research should help in the advancement of healthcare and the tackling of the high burden of disease in those countries [1, 2]. Any research involving human participants should be guided by fundamental ethical principles in order to ensure the protection of their rights.

Advances in research on biological sampling have led to the development of several diagnostic and therapeutic agents. Such advances have also helped in the identification of genetic mutations that may be associated with the increased risk of certain diseases [3]. In biobanks, the process of collecting and storing of harmonized high quality biospecimens and the careful recording of demographic, clinical, genetic and other data facilitate the identification of biomarkers associated with certain cancers, cardiovascular diseases, and neurological disorders [4, 5]. Such biomarkers could help physicians make more sound clinical decisions, leading to early diagnosis, proper determination of prognosis, and the designing of personalized treatment plans [6, 7].

As such, several studies have examined patients’ depth of knowledge of biobanking and their attitudes towards the possible use of their biological samples in biobanking-related activities [8–11]. The public’s willingness to contribute their biological samples for research is relatively high according to several European and American studies [12–14]. Such attitudes might not be generalizable to other countries with dissimilar cultural backgrounds, ethnic or religious makeups and/or degrees of economic development.

Recently, some studies have attempted to examine the willingness of patients as potential participants in some developing countries to contribute their biological samples to biobanking activities. These studies have however come short of examining every possible aspect of this key stakeholder group’s possible perspective. Factors that may influence patients’ willingness to participate in biomedical research include the possible lack of perceived benefits from participation, the degree of availability of information with regards to the nature and methodologies of biobank-based research, and the level of confidence patients have in the integrity of clinical investigators [15, 16].

Several types of biobanks exist, including disease-based, population-based, genetic, commercial, and virtual biobanks [17]. In the past few years, several disease-based biobanks have been established in Egypt [18, 19]. Knowing the attitudes and concerns of those who may be asked to contribute samples to a biobank would help set strategies to improve recruitment efforts and enhance overall trust between researchers and possible participants. Since all biobanks in Egypt are disease-based, the objectives of this study were to determine the degree of knowledge of the Egyptian patients with regards to biobanking, their willingness to contribute samples to biobank-based research, and to detect any possible correlation between knowledge and attitudes. We also aimed at identifying sociodemographic factors associated with these attitudes. We sought to detail the possible factors and fears that may discourage the patient group of stakeholders from participating in biobank-based research, including possible concerns with regards to confidentiality, and the degree with which they perceived personal gain from such research. Information obtained from this preliminary survey can be used to create more focused surveys in the future. The results of this study should also be helpful to individuals involved in research in Egypt as well as other developing countries with similar demographics. The conclusions presented in this study should also help in the development of educational campaigns geared towards addressing patient concerns and misunderstandings. Hopefully, our findings will also help in the development of a set of national ethical biobanking guidelines for Egypt.

## Subjects and methods

### Setting

The target patients were enrolled from three university hospitals in Egypt. The sites were chosen to represent the three main geographical regions of Egypt; the north (Alexandria), the capital or the center (Cairo), and the south (Assiut). Individuals were recruited from the outpatient clinic waiting areas from the Alexandria Main University Hospital, Kasr Al-Aini Hospital,and the Southern Egypt Cancer Institute.

### Recruitment

We recruited adult Egyptian patients (> 18 years) attending the aforementioned outpatient clinics at the time of the administration of the questionnaire. The sample size was determined using the Epi Info 7 software based on the expected probability of positive attitude of patients towards biobanking (83.7%) [20] to achieve 80% power of the study at 95% confidence limits. The calculated sample size was 210 patients (70 from each study setting).

A preliminary phase was conducted to assess validity and reliability of the questionnaire before its wider use. Initially, three Egyptian experts in the field of biomedical research were asked to assess the degree to which items in the questionnaires are relevant and correctly measure the knowledge and attitudes of patients with regards to biobanking. After that, minimal corrections were done. The next step was pretesting of the questionnaire. We included 30 randomly selected patients; 10 from each of the three institutions. They were asked to respond to the questionnaire twice; the two settings conducted 3 weeks apart. Data collected were used to assess internal consistency reliability using Cronbach’s alpha and to check test retest reliability (by computing the intra-class correlation coefficient). The results showed adequate internal consistency reliability (Cronbach’s alpha =0.75). Moreover, the intra-class correlation coefficient was 0.98.

Research coordinators who were trained to communicate the idea of the survey then visited the clinical sites three times a week to recruit patients. While some of these coordinators were public health physicians, others were house officers who received specific training before visiting clinical sites. These research coordinators approached individuals while waiting for their turns to be examined at the outpatient clinics and briefly informed them of the aim of this interview-based study. Individuals who expressed interest were accompanied to a private office at the clinic site and were given further information about the study. Verbal consent, which was then documented in the application, was obtained from those who had agreed to be fully interviewed. Critically ill and apparently easily irritable patients, as well as patients in apparent pain were excluded. We assumed that the psychological condition of these patients at the current moment will affect their answers. Patients with apparent diminished cognitive capacity were also excluded from the study.

Interviews started by giving the participants a simple definition of biobanks and their functions. We tried to communicate a definition that was both scientific and easy to understand. The definition we used is close to BBMRI-ERIC’s definition of a biobank. BBMRI-ERIC defines a biobank as “A place to store all types of human biological samples, such as blood, tissue, cells or DNA. It also stores data related to the samples as well as other biomolecular resources that can be used in health research” [21]. An Arabic version of the following statement was used to communicate the idea of biobanks to patients: “Biobanks are banks where different samples such as blood or tissue samples are stored. Doctors and researchers use these samples in research to find new diagnostic methods or treatments for various diseases, especially those whose treatment is currently difficult, such as cancer.” The entire interview was conducted using colloquial Arabic language. Participants were encouraged to ask for explanations if they did not understand any question.

### Study tool and data collection

The questionnaire was designed to fit our study objectives, and to reflect knowledge gained from relevant literature. The questionnaire was developed in English and then translated into a simplified Arabic version. It consisted of a section for demographic data/participant’s religious affliction, followed by questions measuring knowledge of the term “Biobank”. Next was a set of questions examining attitudes towards participation in biobank-based research, possible concerns regarding patients’/participants’ rights, beliefs regarding potential benefits, possible obstacles to donation, as well as possible fears of sample sharing, and concerns regarding privacy issues. The questions were related to the ethical norms followed in Egypt, especially for protection of the participants’ rights and data. We are providing here three examples for the standard operating procedures (SOP) from the Egyptian National Cancer Institute (ENCI) biobank that ensure that these rights are preserved.

The first SOP is the ENCI biobank SOP about the informed process. The following paragraph is about presenting the informed consent to a subject who can’t read “If the subject/representative cannot read, obtain an impartial witness to be present during the entire consent discussion to attest that the information in the consent form and any other information provided was accurately explained to, and apparently understood by the subject/representative, and that consent was freely given. The witness may be a family member or friend. The witness should not be a person involved in the design, conduct or reporting of the research study.”

The second one is the ENCI biobank SOP about withdrawal of consent. The following paragraph is in the procedure section of the SOP “The participant may withdraw consent at any time. Personnel at the tumor biobank should take appropriate steps to respect the will of the participant and ensure that the participant is able to withdraw without consequence.”

The third SOP is the ENCI biobank SOP about information access control. The following paragraph is in the purpose section of the SOP “ENCI biobank is intended to manage the safekeeping of clinical and sample data in its custody, and it is accountable for limiting disclosure of information, maintaining privacy of the participants and safeguarding the integrity of the information.”

Participants were de-identified using a unique code, which was coded to the individual’s demographic form. A four-month period was required to achieve the required sample size. The data was collected between March and June 2018.

### Statistical analysis

Data was analyzed using SPSS, version 20 [22]. Data was presented as numbers and percentages for categorical variables and means and standard deviations (SD) for continuous variables. For the purpose of testing associations between qualitative variables, the chi-squared Fisher’sexact and Monte Carlo tests were used. All results were interpreted at the 5% level of significance.

## Results

A total of 259 participants agreed to participate in the study; 100 from the Alexandria Main University Hospital, 89 from Kasr Al-Aini Hospital and 70 from the Southern Egypt Cancer Institute. General and sociodemographic data of the studied participants are presented in Table 1.

**Table 1:** Sociodemographic data of studied participants (*n* = 259)

Studied Variables	*N*	%
Age		
Min. – Max.	18–80	
Mean ± SD.	40.12 ± 14.44	
Sex		
Male	107	41.3
Female	152	58.7
Religion		
Muslim	240	92.7
Christian	19	7.3
Education		
Illiterate	66	25.5
Literate	7	2.7
Primary/preparatory school	66	25.5
Secondary school	58	22.4
University	62	23.9

### Knowledge about the term “biobank”

To assess their knowledge of the term “biobank”, participants were first asked if they had ever heard the term “Biobank”. Most participants (81.1%) said that they had never heard the term before. There was a significant association between knowing of biobanks and higher level of education (*p* < 0.001) and with male gender (*p* = 0.012). There was no such association between knowledge of biobanks and age (*p* = 0.203), or religious affiliation (*p* = 0.371). Additional file 1: Table S1 shows the correlation between knowledge of biobanks and sociodemographic characters of studied patients.

### Attitudes towards donating samples for research

We asked the participants several questions regarding their willingness to donate samples for biobank-based research. As shown in Table 2, 85.3% of those interviewed indicated that they would donate samples to biobanks even if they had not done this before, 85.7% indicated they would donate a urine sample, 78.8% were willing to donate blood samples, and 58.3% were willing to participate in a genetics-related research project.

**Table 2:** Attitude of studied participants towards the donation to biobanks (*n* = 259)

Studied Variables	*N*	%
I think I will donate samples to the biobank even if I have not done this before		
Yes	221	85.3
I am not sure	32	12.3
No	6	2.4
If you are asked to donate a urine sample to scientific research, would you agree?		
Yes	222	85.7
No	37	14.3
If you are asked to donate a blood sample of scientific research, would you agree?		
Yes	204	78.8
No	55	21.2
Would you agree to participate in a research project related to heredity and genes?		
Yes	151	58.3
No	108	41.7

The purpose of the first question was to evaluate general attitudes towards sample donation and to correlate between readiness to donate samples and sociodemographic data and with general knowledge of biobanks. There was no significant association between the degree of willingness to donate and any of the sociodemographic data or general knowledge ofbiobanks. These results are detailed in Additional file 1: Table S2.

### Benefits from and obstacles towards donating samples

Several questions were designed to evaluate participants’ beliefs with regards the potential benefits of participation in biobank-based research, and to understand any obstacles that could prevent them from donating biospecimens. In general, most participants believed that there were benefits in participating in medical research and did not list major obstacles that might prevent them from donating. Opinions of participants with regards to the potential benefits and possible obstacles towards donating samples are shown in Additional file 1: Table S3.

### Security, privacy, and access to samples

Upon asking participants about the issue of security and access to samples (see Table 3), most of them (91.1%) expressed the view that the researchers must maintain the privacy and confidentiality of donor information during the course of scientific research, and 64.1% did not think that the samples and data would be used without their consent or in a way that would breach privacy. About 70% did not think that the security of this data could be compromised, or that it could be stolen and used against them. Of those interviewed, 32.4% agreed to allow the sharing of their samples with researchers abroad. Only 27.8% supported giving access to such samples to pharmaceutical companies. On the contrary, as many as 71.8% believed that law enforcement agencies should have an access to biological samples stored at biobanks, whenever they deemed it necessary.

**Table 3:** Opinions of participants about issues related to security, privacy and access to samples in the biobank (*n* = 259)

Studied Variables	*N*	%
Samples donated for scientific research could be used for purposes inconsistent with the will of the donors.		
Agree	71	27.4
Disagree	166	64.1
I am not sure	22	8.5
Researchers must maintain the privacy and confidentiality of donor information during the course of scientific research.		
Yes	236	91.1
No	9	3.5
I am not sure	14	5.4
Samples donated to the biobank may be sent to persons or institution outside the country.		
Yes	84	32.4
No	121	46.7
I am not sure	54	20.8
Samples donated to the biobank can be shared with pharmaceutical companies.		
Yes	72	27.8
No	118	45.6
I am not sure	69	26.6
The police have the right to access the donated samples when necessary.		
Yes	186	71.8
No	37	14.3
I am not sure	36	13.9

### Potential rights of sample donors and the return of research results

When we asked participants what they believed the potential rights of donors of biological samples to be, only about 25% thought donors should receive financial compensation in exchange for donating samples to research, while 51.3% thought that a participant is able to ask to withdraw his samples after participation. About 50% believed that the donor would no longer own the sample anymore after donating it. Concerning the return of research results, 54.8% thought that the results of research conducted on donated samples should appear in the medical records of the participants, and 92.7% believed that the researcher should contact the donor if analysis of his/her samples indicated that he/she may be potentially at risk of developing a certain disease (Table 4).

**Table 4:** opinions of participants regarding the potential rights of sample donors and the return of research results (*n* = 259)

Studied Variables	*N*	%
The donor has the right to receive a financial compensation for the donated sample		
Yes	64	24.7
No	184	71.0
I am not sure	11	4.3
The sample donated to the biobank is no longer the property of the donor after donation.		
Yes	130	50.2
No	99	38.2
I am not sure	30	11.6
The donor has the right to claim his own sample after donation to the biobank or to request to stop participating in the research study after donating his samples.		
Yes	68	26.3
No	133	51.3
I am not sure	58	22.4
The results of the scientific research on the samples should appear in the medical records of the donor.		
Yes	142	54.8
No	49	18.9
I am not sure	68	26.3
The researcher must contact the donor of the samples if the research shows that he/she is at risk of developing a certain disease		
Yes	240	92.7
No	13	5.0
I am not sure	6	2.3

## Discussion

Egypt is one of the biggest countries in Africa, the Middle East as well as the Arab World. Several biobanks have recently been established in Egypt [18, 19]. Although some efforts have been made to familiarize the scientific community with the concept of biobank-based research [19], efforts to introduce biobanks to the general public have been minimal [19]. Establishing and maintaining a good relationship with biobank stakeholders, including patients or potential participants, is essential for the success and social sustainability of biobanks [23]. To establish this relationship, the first step should be the assessment of knowledge and attitude of all stakeholders towards biobanking and biobank-based research.

In the current work, we aimed to determine the degree of knowledge of biobanks among the patients interviewed and to ascertain the effect of such degree of knowledge on their willingness to participate in biobank-based research (through donating samples). We present the results of this survey conducted at three outpatient clinics at three university hospitals in different regions of Egypt. As the term“Biobank” is new in Egypt, we expected that the vast majority would have no prior knowledge of it. Indeed, 81% of participants had never heard of the term before. This limited knowledge is consistent with the results of surveys previously conducted in other parts of the world [9–11]. We think that this limited knowledge is not just related to the novelty of the concept in Egypt, but also because the term itself might be confusing to many people. The prefix “Bio” in the word “Biobank” does not refer exactly to the function of a biobank. In a previous work that attempted to introduce biobanks to undergraduate students of life sciences, they confused the term “Biobank” with stem cell banks, corneal banks, as even sperm banks [19].

There were significantly higher levels of knowledge of the term ‘Biobank’ among males and those with higher level of education than females or the less educated. No such correlation was found between knowledge and either age or religious affiliation (Additional file 1: Table S1). These results make sense, since about 25% of participants in our study were illiterate, making it difficult for them to have proper knowledge of biobanks or their roles as research institutions. Difference in knowledge between men and women could be reflection of the community culture in Egypt, where males are about 4 times more employed than females [24]. This allows them to have more communications and exposure to the external community than women, especially in lower education and socioeconomic sectors. Moreover, according to the 2015 Global Gender Gap Index, which measures disparities between men and women across countries, Egypt ranks at 136 out of 145 countries worldwide, with lower literacy among females (65% literacy for women versus 82% of males) [25]. This is reflected in lower health awareness among women compared to men and decreased general knowledge in general. This data could explain the result of the present study which showed that men had significantly higher knowledge on biobanks than women. These results are quite similar to results published by Ahram et al. in Jordan, who showed a correlation between knowledge of biobanks and higher levels of education, but not with gender or age [10].

### Attitude towards the idea of donating samples and the benefits of donating

The first step in the creation of a successful biobank is the creation of an inventory. This requires the participation of motivated individuals ready to donate biospecimens and offer data [10, 26]. Understanding the attitudes of potential participants towards donating biological samples is a basic step towards the success of any drive to collect samples and data. Although using the terms “donors” and “donating” may indicate gift-giving or charity, we preferred using them since these terms are commonly found in biobank websites in the donor section, and since we thought that using these terms entails and encourages more involvement and sharing rather than the terms “participants” and “participation” for example. Several questions were devised to assess the willingness of our participants to donate samples. We started with a general question, then asked more specific questions about donating a non-invasive sample (urine), a minimally invasive sample (blood), and about participating in a specific type of research (genetic research). A majority of participants in this study (85.3%) were willing to donate samples for the first time. About 85.7% were willing to donate urine samples, and about 79% of them were willing to donate blood samples for research. These results are comparable to those of a previous study, which found that two-thirds of Egyptians surveyed were willing to donate blood samples for future research [27]. In another study, about 97% of Egyptian parents of pediatric cancer patients agreed to donate blood samples for a cancer research biobank [28]. Our results showed similar attitudes towards donating samples for biomedical research to the attitudes of the general public in Germany, [11] Jordan, [10] and Sweden [14]. Although our results are similar to the published literature, we think that the small sample size and the selection bias associated with obtaining information from only those who agreed to participate in this research study is a limitation in our study. We did not ask participants about whether this attitude towards particpation would mean a willingness to give broad consent to the use of samples for several biobank-based research purposes at the same time, or only a specific consent. This represents another limitation of this study, which should be taken into consideration in future specific surveys.

Interestingly, most interviewees in this study believed in the benefits of medical research and participation. The majority of them were not concerned about the specific potential obstacles presented to them (Additional file 1: Table S3). Similar findings of a positive attitude towards donating samples for research were found by Khalil et al. who reported a great trust in medical research among their study sample [29].

Nevertheless, only 58.3% were willing to participate if the research would involve the study of heredity or genomics. This might reflect some concerns our interviewees have with regards to genetic research, where some people may worry about potential discrimination or believe that any such research would be “Playing God”, with manipulation of genes and traits [30]. Our now better understanding of the potential attitudes of the general public towards donating samples to research should help develop and tailor public education programs/campaigns which should tackle potential concerns with respect to biobank-based research [31].

To study the correlation between the attitudes towards donation and sociodemographic data we chose one question “I think I will donate samples to the biobank even if I had not done this before”. While the attitude towards donation was positive in general, we did not find a significant correlation between this attitude and any of the sociodemographic data, including age, or with general knowledge about biobanks (Additional file 1: Table S2). Studies conducted on the effect of sociodemographic data on attitudes towards donating to biobanks have reported contradictory results. For example, while Labib et al. [28] and Nilstun and Hermerén [32] have reported negative or restrictive attitudes towards donating samples to biobanks among those with higher levels of education, Ahram et al. [10] reported the presence of a positive correlation between higher education and willingness to donate samples for research.

### Sample sharing across borders and commercialization

Biobanks collect both human samples and data, including genetic information for use in research that would investigate various diseases [33]. Availability of a large number of samples and sufficient data is becoming increasingly important to the conduction of badly needed research and the acceleration of the rate of discovery of causes and treatments of various diseases [34]. Such needed research usually entails the transfer of samples from developing countries with limited facilities to more developed countries with advanced technologies. This transfer raises many ethical questions about rights and equity.

When we asked our participants if they agree to share their samples with researchers or institutions abroad, only 32.4% agreed. In a previous study, Egyptian patients preferred sample sharing with Arab countries to Western countries [27]. This perhaps reflects more trust of Egyptians in Arab countries which share some aspects of culture as norms, values and religion with Egypt. In the mentioned study, researchers based this preference by pointing to concerns regarding the type of research that might be conducted on their samples, commercialization issues, as well as their religious beliefs [27].

Sample sharing across borders raises social and political debates in Egypt [35]. Recently, a law tackling clinical research was proposed and discussed in the Egyptian Parliament. In the discussed version, the proposed legislation allows sample transportation out of Egypt only after the approval of the county’s security apparatus [36]. Again, this reflects the public concerns as well as the concerns of some policy makers regarding the possible abuse of these samples and data that would be collected from research conducted on them.

Commercialization of samples in biobanks raises ethical concerns regarding fairness and proper sharing of benefits [37]. Commercialization is one of the concerns of any biobank sample donors and represents a key factor that may affect potential participation [33]. About 46% of interviewees in our study did not support sharing their potential samples with pharmaceutical companies (Table 3). These results are not odd since it has been also reported that the involvement of commercial entities is among factors that deter participation in biobank conducted research [30]. In aforementioned proposed legislation regarding medical research, one article would prohibit “trading of human samples used for research” [36]. We believe that fair distribution of benefits can enhance trust and allow more collaboration with these pharmaceutical companies. These benefits may include sharing in authorship in scientific papers, patents and intellectual property rights for researchers participating with their samples in research with commercial entities, as well as providing drugs that may come out of this research at an affordable price for the public.

Although we did not directly ask about data sharing, we think that our results reflect the fears and concerns of our study participants about the possible misuse of their data if they were to be shared with specific entities. Since there is limited data about public attitudes toward data sharing from the Arab World, [38] we think that our data contributes to the current literature in this respect. However, this data is not generalizable to other countries in the region and data from each country should be collected and analyzed individually.

### Security and confidentiality, and access to samples

Biobanks collect sensitive information about participants who consent to donating their samples and data for a broad range of research purposes. This data includes clinical, laboratory as well as genetic data [31]. Biobanks should thus have and follow documented procedures to protect sample donors from any violation of their privacy and to ensure that samples and data would only be used in a way consistent with the consent collected from donors [39]. Protection of privacy are among the factors that affect potential participation in biobanks [40].

Asking participants about issueof security and use of samples (Table 3), we found that most of them were concerned about issues of security, and did not think that samples and data should be used against their will. Our interviewees’ concerns reflect a view detailed in the current Egyptian Constitution which stipulates in article number 60 that “No medical or scientific experiment may be performed without the citizen’s free and documented consent and in accordance with established principles” [41]. A survey conducted among a range of groups in Saudi Arabia reported similar concerns, with most participants voicing the importance of confidentiality in biobanks [42].

Interestingly, 71.8% of participants thought that law enforcement should be able to have an access to donated samples and data whenever they deemed it necessary. In a previous study conducted in Egypt, most participants thought that the government should approve any transport of samples abroad [27]. This possibly reflects a preference among Egyptians that medical research be conducted under a level of government oversight. This might not be the situation in other countries, and thus should be taken into consideration during any communication with the public about biobank-based research.

### Potential rights of sample donors and return of research results

Ethical guidelines show a considerable debate about who owns research and biobank samples [43]. Some authors proposed that “custodianship” and “stewardship” are the suitable terms to define the role of researchers and the biobanks with regard to samples [44]. However, these guidelines state that research subjects or participants can withdraw their consent at any time [45]. There was quite limited knowledge about these points among our study group (Table 4). Again, this reflects the need for the development of educational programs that inform the general public of rights of participants in medical research, including biobanks.

The return of research results is also a point of contentious ethical debate and controversy within the biobanking community [46, 47]. At issue are such questions as how participants would be affected by the inclusion of information regarding their health and potential future illnesses on access to employment, insurance or proper healthcare [48]. We asked our interviewees two questions regarding this issue. As shown in Table 4, most participants assumed that return of research results to be one of their rights. These results are quite similar to the results of surveys conducted in Saudi Arabia and in Australia, where participants believed that sample donors should be informed about any results that may benefit them [49, 50]. Dealing with this issue is not easy, since researchers and physicians may not know exactly what and how to communicate research results with participants. We think that the development and communication of national ethical biobanking guidelines in Egypt can help settle these questions and give guidance to medical researchers.

### Religious affiliation and attitude towards donation

There is currently only limited research in the literature about the association between religious affiliation and attitudes towards donating samples and giving data to biobanks [31]. The second article of the Egyptian constitution states that “Islam is the state religion, and that principles of Islamic Jurisprudence are the main source of legislation” [41]. The two main religions in Egypt are Islam (mainly Sunni), followed by Christianity (mainly Orthodox). In general, persons of either faiths are religious people who seek religious guidance in matters pertaining to their daily lives, and worry about what their religions allow and not allow [40]. Muslim religious scholars have indicated that Islam allows for the establishment of research biobanks and places a high value on the principle of autonomy and confidentiality [51, 52]. On the other hand, we could not find resources that document the opinion of Christian scholars with regards to the establishment of biobanks.

Religious and cultural debates have previously derailed the establishment of a national organ donation program from deceased donors in Egypt [53]. Fortunately, a similar debate has not risen with regards to biobank-related research and our study participants did not confuse between research sample donation and organ donation. The majority of our participants (86.5%) believed that donating samples for research did not contradict their religious beliefs. Moreover, 93.4% thought that donating samples is a form of charity. In Jordan, more than 60% of participants believed that their religion permitted sample donation for research and expressed that this had a positive effect on their decision to donate [39]. On the other hand, our results are contradictory to the results of the survey conducted in the US, where more religious participants were less willing to donate samples to biobanks [30].

## Conclusion and recommendations

Although there is limited knowledge about biobanking among Egyptians, there is a general positive attitude towards sample donation and no specific cultural or religious barriers against it according to our findings. However, Egyptians have concerns regarding participation in genetic research and regarding sharing their samples across borders or with pharmaceutical companies. Taking it all together, planning and implementation of a research awareness campaign about biobanking aimed at patients attending health care facilities would potentially yield positive results. More specific surveys may be needed in the future to plan for such campaigns, which should take into consideration both the peculiarities of Egyptian culture and the legal framework in Egypt.

## Additional file

Additional file 1: **Table S1.** Correlation between knowledge about biobanks and sociodemographic data of studied patients (*n* = 259). **Table S2.** Attitude towards sample donation to biobanks in relation to sociodemographic data and knowledge of studied patients about the term “Bioabank” (*n* = 259). **Table S3.** Opinions of studied patients regarding the benefits and obstacles of sample donation (*n* = 259). (DOCX 26 kb)

## Data Availability

The datasets used and/or analysed during the current study are available from the corresponding author on reasonable request.

## Abbreviations

BBMRI-ERIC: Biobanking and BioMolecular resources Research Infrastructure-European Research Infrastructure Consortium
ENCI biobank: The Egyptian National Cancer Institute biobank
SOP: Standard operating procedures

## References

[1] Shapiro HT, Meslin EM (2001). Ethical issues in the design and conduct of clinical trialsin developing countries. N Engl J Med.

[2] Benatar SR (2002). Reflections and recommendations on research ethics in developing countries. Soc Sci Med.

[3] Hirtzlin I, Dubreuil C, Prèaubert N (2003). An empirical on biobanking of human genetic material and data in six EU countries. Eur J Hum Genet.

[4] Schroeder D, Lasen-Diaz C (2006). Sharing the benefits of genetic resources: from biodiversity to human genetics. Dev World Bioeth.

[5] Feero WG, Guttmacher AE, Collins FS (2010). Genomic medicine, an updated primer. N Engl J Med.

[6] Ollier W, Sprosen T, Peakman T (2005). UK biobank: from concept to reality. Pharmacogenomics.

[7] Hewitt RE (2011). Biobanking: the foundation of personalized medicine. Curr Opin Oncol.

[8] Madsen SM, Mirza MR, Holm S (2002). Attitudes towards clinical research amongst participants and nonparticipants. J Intern Med.

[9] Chen H, Gottweis H, Starkbaum J (2013). Public perceptions of biobanks in China: a focus group study. Biopreserv Biobank.

[10] Ahram M, Othman A, Shahrouri M (2012). Public perception towards biobanking in Jordan. Biopreserv Biobank.

[11] Bossert S, Kahrass H, Strech D (2018). The public’s awareness of and attitude toward research biobanks - a regional German survey. Front Genet.

[12] Kass NE, Maman S, Atkinson J (2005). Motivations, understanding, and voluntariness in international randomized trials. IRB.

[13] Cousins G, McGee H, Ring L (2005). Public perceptions of biomedical research: a survey of the general population in Ireland.

[14] Kettis-Lindblad A, Ring L, Viberth E (2006). Genetic research and donation of tissue samples to biobanks. What do potential sample donors in the Swedish general public think?. Eur J Pub Health.

[15] Bauer K, Taub S, Parsi K (2004). Ethical issues in tissue banking for research: a brief review of existing organizational policies. Theor Med Bioeth.

[16] Maschke KJ, Murray TH (2004). Ethical issues in tissue banking for research: the prospects and pitfalls of setting international standards. Theor Med Bioeth.

[17] De Souza YG, Greenspan JS (2013). Biobanking past, present and future: responsibilities and benefits. AIDS.

[18] Labib RM, Mostafa MM, Alfaar AS (2016). Biorepository for pediatric Cancerwith minimal resources. Biopreserv Biobank.

[19] Abdelhafiz AS, Fouda MA, El-Jaafary SI (2017). Targeting future customers:an introductory biobanking course for undergraduate students of life sciences. Biopreserv Biobank.

[20] Hassona Y, Ahram M, Odeh N (2016). Factors influencing dental PatientParticipation in biobanking and biomedical research. Med Princ Pract.

[21] BBMRI-ERIC website Available at: http://www.bbmri-eric.eu/about/ (Accessed 24 June 2019).

[22] IBM Corp. Released 2011. IBM SPSS statistics for windows, version 20.0. Armonk: IBM Corp.

[23] Simeon-Dubach D, Henderson MK (2014). Sustainability in biobanking. Biopreserv Biobank.

[24] The Egyptian Central Agency for Public Mobilization and Statistics Statistical Year book. Available at: https://www.capmas.gov.eg/Pages/StaticPages.aspx?page_id=5034 (Accessed 24 Jun 2019).

[25] The Global Gender Gap Index 2015. Available at http://reports.weforum.org/global-gender-gap-report-2015/the-global-gender-gap-index-2015/ (Accessed 24 Jun 2019).

[26] Critchley CR, Nicol D, Otlowski MF (2012). Predicting intention to biobank: a national survey. Eur J Pub Health.

[27] Abou-Zeid A, Silverman H, Shehata M (2010). Collection, storage and use ofblood samples for future research: views of Egyptian patients expressed in a cross sectional survey. J Med Ethics.

[28] Labib RM, Hassanain O, Alaa M (2018). Planning today for Tomorrow’s research: analysis of factors influencing participation in a pediatric cancer research biorepository. Front Oncol.

[29] Khalil SS, Silverman HJ, Raafat M (2007). Attitudes, understanding, and concerns regarding medical research amongst Egyptians: a qualitative pilot study. BMC Med Ethics.

[30] Johnsson L, Helgesson G, Rafnar T (2010). Hypothetical and factual willingness to participate in biobank research. Eur J Hum Genet.

[31] Sanderson SC, Brothers KB, Mercaldo ND (2017). Public attitudes toward consent and data sharing in biobank research: a large multi-site experimental survey in the US. Am J Hum Genet.

[32] Nilstun T, Hermerén G (2006). Human tissue samples and ethics-attitudes of the general public in Sweden to biobank research. Med Health Care Philos.

[33] Nicol D, Critchley C, McWhirter R (2016). Understanding public reactions to commercialization of biobanks and use of biobank resources. Soc Sci Med.

[34] Sariyar M, Schluender I, Smee C (2015). Sharing and reuse of sensitive data and samples: supporting researchers in identifying ethical and legal requirements. Biopreserv Biobank.

[35] The president objects to the law of clinical medical research. Available at https://www.almasryalyoum.com/news/details/1328846. (Accessed 24 Jun 2019).

[36] The proposed law of clinical medical research. Available at https://bit.ly/2EB6va8 (Accessed 24 Jun 2019).

[37] Cambon-Thomsen A, Rial-Sebbag E, Knoppers BM (2007). Trends in ethical and legal frameworks for the use of human biobanks. EurRespir J.

[38] Howe N, Giles E, Newbury-Birch D (2018). Systematic review of participants' attitudes towards data sharing: a thematic synthesis. J Health Serv Res Policy.

[39] Campbell LD, Betsou F, Garcia DL (2012). Best practices for repositories collection, storage, retrieval, and distribution of biological materials for research biobanking. Biopreserv Biobank.

[40] Ahram M, Othman A, Shahrouri M (2013). Factors influencing public participation in biobanking. Eur J Hum Genet.

[41] The Egyptian Constitution project website. Available at https://www.constituteproject.org/constitution/Egypt_2014.pdf?lang=ar. (Accessed 24 Jun 2019).

[42] Alahmad G, Hifnawy T, Abbasi B (2016). Attitudes toward medical and genetic confidentiality in the Saudi research biobank: an exploratory survey. Int J MedInform.

[43] Cadigan RJ, Easter MM, Dobson AW (2011). "That's a good question": university researchers' views on ownership and retention of human genetic specimens. Genet Med.

[44] Hens K, Nys H, Cassiman JJ, Dierickx K (2009). Genetic research on stored tissue samples from minors: a systematic review of the ethical literature. Am J Med Genet A.

[45] Helgesson G, Johnsson L (2005). The right to withdraw consent to research on biobank samples. Med Health Care Philos.

[46] Bledsoe MJ, Clayton EW, McGuire AL (2013). Return of research results from genomic biobanks: cost matters. Genet Med.

[47] De Clercq E, Kaye J, Wolf SM (2017). Returning results in biobank research: global trends and solutions. Genet Test Mol Biomarkers.

[48] Bledsoe MJ, Grizzle WE, Clark BJ (2012). Practical implementation issues and challenges for biobanks inthe return of individual research results. Genet Med.

[49] Alahmad G, Dierickx K (2017). Return of research results in the Saudi biobank: an exploratory survey. Genet Test Mol Biomarkers.

[50] Glass DC, Kelsall HL, Slegers C, et al. A telephone survey of factors affecting willingness to participate in health research surveys. BMC Public Health. 2015;15(1). 10.1186/s12889-015-2350-9. 10.1186/s12889-015-2350-9 PMC4594742 26438148

[51] Alahmad G, Dierickx K (2012). What do Islamic institutional fatwas say about medical and research confidentiality and breach of confidentiality?. Dev World Bioeth.

[52] Alahmad Ghiath, Dierickx Kris (2018). Ethics of Research Biobanks: Islamic Perspectives. Biopreservation and Biobanking.

[53] Hamdy S (2013). Not quite dead: why Egyptian doctors refuse the diagnosis of death byneurological criteria. Theor Med Bioeth.

