# Supplementary material for: What Egyptians think. Knowledge, attitude, and opinions of Egyptian patients towards biobanking issues
Source: BMC Med Ethics. 2019 Aug 9;20:57. doi: 10.1186/s12910-019-0394-6 (PMC6689171; doi:10.1186/s12910-019-0394-6)
Supplement: Supplementary file 1 — Table S1. Correlation between knowledge about biobanks and sociodemographic data of studied patients (n = 259). Table S2. Attitude towards sample donation to biobanks in relation to sociodemographic data and knowledge of studied patients about the term “Bioabank” (n = 259). Table S3. Opinions of studied patients regarding the benefits and obstacles of sample donation (n = 259). (DOCX 26 kb) [file 12910_2019_394_MOESM1_ESM.docx]

**Additional file 1**

**Table S1 Correlation between knowledge about biobanks and sociodemographic data of studied patients (n=259)**

| Studied Variables | Heard about biobanks | | | | Test of sig. | p |
| --- | --- | --- | --- | --- | --- | --- |
|  | Yes  (n=49) | | No  (n=210) | |  |  |
|  | No. | % | No. | % |  |  |
| Age |  |  |  |  | χ^2^=4.601 | 0.203 |
| < 20 | 5 | 10.2 | 10 | 4.8 |  |  |
| 20- < 40 | 23 | 46.9 | 89 | 42.4 |  |  |
| 40- < 60 | 19 | 38.8 | 86 | 41.0 |  |  |
| ≥ 60 | 2 | 4.1 | 25 | 11.9 |  |  |
| Sex |  |  |  |  | χ^2^=6.246^*^ | 0.012^*^ |
| Male | 28 | 57.1 | 79 | 37.6 |  |  |
| Female | 21 | 42.9 | 131 | 62.4 |  |  |
| Religion |  |  |  |  | χ^2^=0.731 | ^FE^P=0.371 |
| Muslim | 44 | 89.8 | 196 | 93.3 |  |  |
| Christian | 5 | 10.2 | 14 | 6.7 |  |  |
| Education |  |  |  |  | χ^2^=24.632^*^ | <0.001^*^ |
| Illiterate/read and write | 0 | 0.0 | 73 | 34.8 |  |  |
| Primary/preparatory | 18 | 36.7 | 48 | 22.9 |  |  |
| Secondary | 13 | 26.5 | 45 | 21.4 |  |  |
| University | 18 | 36.7 | 44 | 21.0 |  |  |

t: Calculated value of the Student t test

χ^2^: Calculated value for Chi-square test

^FE^P: P-value of Fisher’s Exact test

*: Statistically significant at p ≤ 0.05

**Table S2 Attitude towards sample donation to biobanks in relation to sociodemographic data and knowledge of studied patients about the term “ Bioabank” (n=259)**

| Studied Variables | Willing to donate | | | | | | Test of sig. | p |
| --- | --- | --- | --- | --- | --- | --- | --- | --- |
|  | Yes  (n=221) | | | Yes  (n=221) | | |  |  |
|  | No. | | % | No. | | % |  |  |
| Age |  | |  |  | |  | χ^2^=3.663 | ^MC^P=0.296 |
| < 20 | 14 | | 6.3 | 1 | | 2.6 |  |  |
| 20- < 40 | 96 | | 43.4 | 16 | | 42.1 |  |  |
| 40- < 60 | 91 | | 41.2 | 14 | | 36.8 |  |  |
| ≥ 60 | 20 | | 9.0 | 7 | | 18.4 |  |  |
| Sex |  |  | |  |  | | χ^2^=1.740 | 0.187 |
| Male | 95 | 43.0 | | 12 | 31.6 | |  |  |
| Female | 126 | 57.0 | | 26 | 68.4 | |  |  |
| Religion |  |  | |  |  | | χ^2^=0.020 | ^FE^P=0.747 |
| Muslim | 205 | 92.8 | | 35 | 92.1 | |  |  |
| Christian | 16 | 7.2 | | 3 | 7.9 | |  |  |
| Education |  |  | |  |  | | χ^2^=2.606 | 0.456 |
| Illiterate/read and write | 63 | 28.5 | | 10 | 26.3 | |  |  |
| Primary/preparatory | 59 | 26.7 | | 7 | 18.4 | |  |  |
| Secondary | 46 | 20.8 | | 12 | 31.6 | |  |  |
| University | 53 | 24.0 | | 9 | 23.7 | |  |  |
| Have you ever heard about biobanks? |  |  | |  |  | | χ^2^=0.964 | 0.326 |
| Yes | 44 | 19.9 | | 5 | 13.2 | |  |  |
| No | 177 | 80.1 | | 33 | 86.8 | |  |  |

**Table S3 Opinions of studied patients regarding the benefits and obstacles of sample donation (n=259)**

| **Studied Variables** | **n** | **%** |
| --- | --- | --- |
| **Donating samples for scientific research helps to prevent or treat diseases.** |  |  |
| Agree | 228 | 88.0 |
| Disagree | 8 | 3.1 |
| I am not sure | 23 | 8.9 |
| **Donating samples for scientific research wastes the time of the donor.** |  |  |
| Agree | 43 | 16.6 |
| Disagree | 201 | 77.6 |
| I am not sure | 15 | 5.8 |
| **If I do not donate samples for scientific research, this might affect my right to getting proper health care.** |  |  |
| Agree | 52 | 20.1 |
| Disagree | 193 | 74.5 |
| I am not sure | 14 | 5.4 |
| **If I donate today to scientific research, this may benefit me in the future.** |  |  |
| Agree | 238 | 91.9 |
| Disagree | 7 | 2.7 |
| I am not sure | 14 | 5.4 |
| **If I donate my samples today for scientific research, this may benefit my family in the future.** |  |  |
| Agree | 230 | 88.8 |
| Disagree | 9 | 3.5 |
| I am not sure | 20 | 7.7 |
| **Donating samples for scientific research causes a financial burden on the donor** |  |  |
| Yes | 36 | 13.9 |
| No | 180 | 69.5 |
| I am not sure | 43 | 16.6 |
